# General Practitioners practice nurses and parents’ perspectives on childhood overweight management – a qualitative study

**DOI:** 10.1080/13814788.2024.2402259

**Published:** 2024-09-27

**Authors:** Maxime Adriana Maria van der Velden, Hevy Hassan, Dieuwke Schiphof, Madelon van Tilborg-den Boeft, Sylvia Buis, Wilma Jansen, Patrick Jan Eugène Bindels, Marienke van Middelkoop

**Affiliations:** a Department of General Practice, Erasmus MC Medical University Center Rotterdam, The Netherlands; b Department of Public Health, Erasmus MC Medical University Center Rotterdam, The Netherlands; c Department of Social Development, City of Rotterdam, The Netherlands

**Keywords:** Children, overweight, obesity, screening, qualitative research

## Abstract

**Background:**

Overweight and obesity in children is a major health problem. General practice might be a promising setting for identifying and for the first steps in the management of overweight and obesity in children.

**Objective:**

To explore opinions, needs and preferences about the role of general practice in the management of overweight and obesity in children from the perspectives of Dutch general practitioners (GPs), practice nurses (PNs) and parents of children with and without overweight.

**Methods:**

A qualitative study using semi-structured focus group interviews. GPs and PNs were recruited from general practices from the region South-Western. Parents were mainly recruited via social media and primary schools. Twenty-five GPs, seven PNs and 18 parents were interviewed. All interviews were audio recorded, transcribed and thematically analysed.

**Results:**

GPs, PNs and parents agreed that it is the task of the GP to identify, address and refer children with overweight and obesity. However, GPs find it difficult to start this conversation due to time constraints; fear for the reaction of parents and children; lack of clarity about treatment and referral options. Parents indicated they are open to a conversation if the GP is non-judgmental, honest and respectful. PNs saw no role in managing overweight and obesity in children.

**Conclusion:**

Although GPs experience several barriers, GPs, PNs and parents all agreed that GPs should play a role in identifying, addressing and referring children with overweight and obesity. Supportive tools are required for GPs in order to play this role.

## KEY MESSAGES

General practitioners recognise that they can play an important role in the management of overweight and obesity in children.

Parents are open for weight-related conversations if general practitioners are non-judgemental and have an open attitude.

General practitioners need supportive tools to identify, address and refer children with overweight and obesity.

## Introduction

Childhood overweight and obesity are an increasing health problem worldwide [1,2]. In the Netherlands, 11.5% of the children aged four to 12 years have overweight [3,4]. Children with overweight have an increased risk to develop health problems like high cholesterol and hypertension [5,6]. In addition, these children are more likely to become overweight adults with an increased risk to develop diseases such as cardiovascular diseases, type 2 diabetes and diverse types of cancer [7]. Next to that, children with overweight experience more psychosocial problems compared to children with a normal body weight, such as low self-esteem, depression and they are more often victims of bullying [8]. Hence, it is imperative to identify, address and treat overweight and obesity in children as early as possible [9,10].

In the Netherlands, the general practitioner (GP) is the first point of care for health complaints. Most ­children visit their GP at least once during each life year [5,11]. This makes general practice a promising setting to identify and address overweight and obesity in children [9,11,12]. The Dutch Obesity guideline for GPs states that obesity in children should always be addressed, no matter the reason of consultation and recommends measuring bodyweight and height of children suspected to have overweight or obesity [13–15]. However, it appears that most Dutch GPs do not adhere to these guidelines, since only 24% of them determine the Body Mass Index (BMI) of children who seem to be overweight and the number of GPs who refer these children is even lower [1,3,13,16]. This is likely related to barriers experienced by GPs in clinical practice, such as sensitivity of the topic, lack of time and fear of disturbing the doctor-patient relationship [7,9,10,13,17]. Moreover, current guidelines do not seem to offer enough support to identify and discuss overweight and obesity in children in general practice [14]. It is therefore especially important to get a better understanding of the opinions and needs of GPs in managing overweight and obesity in children.

Next to GPs, also practice nurses (PNs) working in general practice could be involved in the management of overweight and obesity in children. Based on previous literature, a cooperation between GPs and PNs might be possible by expanding overweight management tasks to PNs [9,17,18]. However, little is known about their willingness and needs to play a role in this management. Furthermore, in order to manage overweight and obesity in children in general practice, it is crucial to explore the expectations and preferences of parents regarding the role of general practices in tackling overweight and obesity in children.

Therefore, the primary aim of this study was to gather insights into the opinions, needs and preferences regarding the role of general practice in the management of overweight and obesity in children from the perspectives of GPs, PNs and parents.

## Methods

### Study design

A qualitative study was set up to investigate opinions, needs and preferences about the role of general practice in the management of overweight and obesity in children. This was done from three perspectives: GPs, PNs and parents of children with and without overweight or obesity.

Semi-structured focus-group interviews were conducted using an interview guide [Appendix 1A1-3, supplementary material]. The topics and open-ended questions of the interview guide were developed based on literature, the Dutch GP guideline Obesity [14], and the report ‘the Minimal Intervention Strategy (MIS) for adult overweight’ [19]. The following themes were chosen for the interviews with GPs, PNs and parents: expectations and preferences about the role of general practice in identifying and addressing overweight and obesity in children, ways to initiate the conversation and to discuss overweight and obesity, treatment and referral options and information materials. The interview guides were pilot-tested with two GP trainees, two researchers and one parent which led to minor changes. During the study period no adaptations to the interview guides were made. Group interviews were performed as this allows interaction between participants which provides more relevant and in-depth information about their opinions, experiences and differences in perspectives about this topic than individual interviews.

### Recruitment of participants and sampling

GPs and PNs were recruited in the region of Rotterdam by email using the Academic Network of General Practitioners of the Erasmus MC University Medical Centre, a newsletter was sent by email to 376 GP trainers and a recruitment call was placed on the page of the Dutch Association of Practice Nurses. All participating GPs and PNs had to fulfil the following inclusion criteria: currently working as GP or PNs for at least two days a week in a general practice.

Parents were recruited using a previously conducted cohort study [20], social media, primary schools in the Rotterdam region and the obesity clinic at Sophia Children’s Hospital in Rotterdam. Parents had to fulfil the following inclusion criteria: having a child aged 2–14 years, with or without overweight or obesity [21], understand and speak the Dutch language.

Thirty-five GPs, 10 PNs and 23 parents showed interest to participate. Interested persons were contacted by the research team by email or phone to inform them about the study. Based on everyone’s availability, focus group interviews were planned per level (GPs; PNs; parents). Before the interviews took place all participants provided an information letter and written informed consent [Appendix 1B-C, supplementary material].

## Data collection

The focus group interviews were conducted at the department of the General Practice in Erasmus MC or online via MS Teams between October 2022 and December 2022. One focus group with parents was performed at a primary school. Each focus group lasted approximately 60 min and all were conducted in Dutch. Two researchers attended all focus groups, with one leading the discussion (MV) and one as observant (HH). All interviews were audio recorded and transcribed verbatim. Transcripts were not returned to participants.

Demographics of participants were collected via an online questionnaire. For GPs and PNs the questionnaire informed on work-related factors and for parents informed about their experiences with overweight management strategies for children [Appendix 1D1-2, supplementary material].

## Data analysis

A phenomenological approach was used for the analysis to emerge new themes since this method allows to explore individual experiences [22]. After each focus group interview a debriefing was held between the interviewer (MV) and the observant (HH) to combine detailed notes. The transcripts were independently coded using MAXqda by two researchers (MV;HH) and were thematically analysed. The codes were categorised into a hierarchy of main themes and subthemes related to the research questions (Table 1). The emerging code trees were reviewed and discussed during regular team meetings (MM;MV;HH;DS;MT). These meetings continued until consensus about theoretical data saturation was reached and when the final themes were perceived to capture and reflected the entire dataset. The consolidated criteria for reporting qualitative research (COREQ) statement was used to inform reporting of the findings [Appendix 2, supplementary material].

**Table 1.** Overview of the themes and subthemes discussed during the interviews.

Themes	Subthemes	Examples
Identifying and addressing overweight and obesity in children	Role of GP and/or PN	e.g. why or why not, recognise overweight, task other organisation/specialist
	Ways to identify and address	e.g. weight and height measurement, link with health complaint
	Barriers to identify and address	e.g. time, request of help, doctor-patient relationship
	Facilitators to identify and address	e.g. parent starts about weight, identified by someone else, positive experience
Starting and continuing a conversation about overweight and obesity	Barriers to start and continue a conversation	e.g. time, lack skills and knowledge GP, reaction of parents and children, sensitive topic
	Facilitators (ways) to start and continue a conversation	e.g. ask permission, ask general questions, tone and word choice
	Bodyweight and height measurement	e.g. BMI values children
Treatment	Referral	e.g. lack of clarity referral options, resources
	Follow-up	e.g. supports patient, interest, logistics, other organisation
	Information resources	e.g. websites, folders

GP: General Practitioner; PN: Practice Nurse; BMI: Body Mass Index.

## Results

A total of eleven focus group interviews were conducted: five with GPs (*N* = 25, group size: 3-7), two with PNs (*N* = 7, group size: 3-4) and four with parents (*N* = 18, group size: 4-5). Demographics of the participants are presented in Table 2.

**Table 2.** Demographics of the participants.

	GPs	PNs	Parents
	(*N* = 25)^a^	(*N* = 7)	(*N* = 18)
Women (%)	44.4%	100%	83.3%
Age (years)	45.2 (11.0)	48.3 (9.5)	41.2 (6.4)
BMI (kg/m^2^)	23.3 (2.5)	21.8 (2.0)	27.4 (6.7)
Work experience (years, months)	14.0 (10.6), 4.7 (3.5)	9.0 (9.5), 6.0 (3.4)	
Work area (urban/non-urban)	17/6	6/1	
Number of children			2.7 (0.96)
Age of children (years)			9.4 (5.9)
Child with overweight (yes/no)			5/13
Experience treating overweight of child (yes/no)			4/14
Nationality			
Dutch			13
Moroccan			3
Surinamese			1
Turkish			1
Antillean			1

GPs: General Practitioners; PNs: Practice Nurses; kg: Kilograms.

Data are presented as mean and SD, unless otherwise stated.

^a^ Missing values of the participated GPs, *n* = 2; did not fill in the online questionnaire.

## Theme 1: Identifying and addressing overweight and obesity in children

GPs recognised that they could play a role in identifying and addressing overweight and obesity in children when the child has conspicuous overweight or obesity, even when the child initially comes for another health complaint: *‘I think it is the role of GPs to identify it. In any case to name it and then ask parents to talk about it’.* (FG4GP3) *‘I agree, I certainly think it is our role, even if the child does not come for it’.* (FG4GP4)

Also parents perceived that GPs have the task to identify and address overweight and obesity in children when it is related to the request for help, but also when children consult for another health complaint. Parents viewed the GP as the right person to identify and address it because of the longitudinal doctor-patient relationship and parents are convinced that GPs have the right medical knowledge and skills: *‘I am in favour that GPs should identify and discuss it. Even if you visit general practice for something else, I think that being overweight should be a signal for GPs. And I think that GPs have the right knowledge and training to do it’.* (FG1P3-KO-OO)

Since children are not the primary focus of the participated PNs, none of them saw a role for themselves in identifying nor addressing overweight and obesity in children. They noted that it is a task for GPs and suggested that it could be a role for PNs specialised in youth, since they work with children more often: *‘We do not see any children, GPs and Youth PNs could play a role in identifying, but we hardly’.* (FG2PN2)

Both GPs and PNs expressed that they find it a task for schools, Dutch youth care institutes and child consultation clinics to identify and address excessive weight in children: *‘I think that in the first instance the largest role is assigned to the consultation clinic or school doctor, because they see children more often’.* (FG2POH3).

## Theme 2: Initiating and continuing a conversation about overweight and obesity

### Barriers

Various barriers to initiate and to continue weight-related conversations were expressed by GPs: consultation time is too short to address overweight on top of handling the request for help: *‘I think you often observe overweight, but you do not always do something because you do not have time for it’.* (FG5GP2)*;* observed overweight is hard to link with the request for help: *‘I certainly think GPs play a role, even if the child does not come for it. But I find it really difficult to address it when the child comes for a completely different health problem’.* (FG4GP4); a proportion of GPs do not like discussing overweight since it is not easy to solve and they do not know what to offer: ‘*Honestly, I do not like it to be ready with my shotgun and to address parents, I would much rather have a child with asthma as for that it is clear how I can help’.* (FG2GP3)*;* fear to disturb the doctor-patient relationship because GPs are afraid for the reaction of parents and children since it is a sensitive topic. For example, GPs mentioned their fear to negatively affect children’s body image or to trigger an eating disorder: *‘I am concerned about the effect of suddenly mentioning overweight when there is no question at all as during that age a negative body image or eating disorder can grow when children realise: ‘the doctor thinks I am too fat’. I think that is the only thing that leaves the consultation room. That makes that I find it a difficult issue’.* (FG2GP1)

Contrary to the mentioned barriers by GPs, all parents indicated that they are open for a conversation about overweight in their child, as long as the GP is non-judgmental, honest, respectful and shows interest: ‘*Indeed being non-judgmental. Just ask general questions about how things are going, do not immediately assume that it will be pizza every day’.* (FG1P2-KO-OO). All parents emphasised that the tone, choice of words and attitude of GPs are especially important for how parents will react. For example, a parent of a child with overweight had a negative experience with a youth doctor who responded judgmentally and pedantically when the child’s overweight was mentioned. This experience left the parent feeling scared, hurt and guilty, and it was perceived as interference, causing irritation: *‘The tone is so important, in my case it was quite intense. My daughter was very young…they said ‘No, this cannot be, she is way too fat, she has morbid obesity, she needs to go to a paediatrician now’. I waited a year before taking further steps, so it did have an effect, but I did not like the way it was addressed’.* (FG3P4-KO-OO)

### Facilitators

The following facilitators were noted by GPs to initiate and continue weight-related conversations: observed overweight is related with the request for help: *‘If they come for example with a knee complaint, then of course you have a way to start about weight’* (FG5GP3); overweight is a problem in the family: *‘It is often also a trend that comes back in the family. Usually, it is not one child but all children or parents, and then I think it helps to address it once’.* (FG1GP6).

GPs and parents suggested similar ways to initiate and continue a conversation about overweight: ask permission whether parents are open for a conversation; ask in-depth questions about the family’s lifestyle; inform about health consequences. Divergent opinions around whether this conversation should take place within the presence of the child. Some parents indicated they would rather tell it themselves because they are afraid that it can hurt their child or make them insecure. Whereas other parents, of which most had a child with overweight, indicated that GPs should involve the child in this conversation by asking questions to the child, because they think that the GP is the right person to explain children the importance of a healthy bodyweight: *‘Just by asking questions to the child itself. For example: ‘Do you have a nice life? How is it going at school? You have grown considerably and become firm, what do you think of this?’ My son thought the questions were logical and not annoying and answered everything’.* (FG1P2-KO-OO) In addition, PNs suggested to use information materials that could support GPs to further inform parents. Both GPs and parents stated that the need for information materials is personal.

### Measure bodyweight and height

Bodyweight and height measurement was seen as a conversation starter by GPs since this prevents fat-shaming: ‘*I do measure bodyweight and height [of children with overweight] on purpose because I want to avoid any kind of fat-shaming’. From ‘oh I can already see that you have overweight’.* (FG1GP3) Both GPs, PNs and some parents shared the opinion that this measurement could provide parents a better insight in the seriousness of the overweight and that it should not take any extra consultation time since it is often already done to prescribe medication: ‘*I think it is fine if a GP measures weight and height of my child…indeed when medications are prescribed or when the GP thinks it would be good to do so that you have some facts to start the conversation’.* (FG3P2-KG-OO)

Conversely, some GPs and parents wondered if it is feasible to measure bodyweight and height within the consultation due to time constraints and lack of measurement materials. Furthermore, other parents would rather not have that this measurement take place when the child is too ill and when it is not necessary to handle the health issue at that moment. Accordingly, some GPs wondered how to explain this measurement when it is not needed to handle the request for help and because it is already done at consultation clinics. Moreover, GPs indicated lack of clarity about BMI-values of children and desire an easy-to-use BMI-calculator: ‘*It helps to make children and parents aware of obesity. But I think we are repeating the work of the Centre for Youth and Family’.* (FG1GP5)

### Theme 3: Treatment of children with overweight and obesity

Both GPs and parents did not see an active role for GPs in the treatment or follow-up policy to tackle overweight and obesity in children because GPs do not have time nor the expertise to guide families. GPs noted that specialists such as dieticians or lifestyle coaches are more suitable to perform this task. GPs and parents agreed that GPs could play a role in informing, advising and referring: *‘I really think it is our job to identify it and to inform them and to point them in the right direction. But I am not going to supervise them and I do not see the treatment within general practice’.* (FG1GP3) However, GPs indicated that they are unaware about available interventions and referral options for these children and stated that an overview would be helpful: *‘I honestly have no idea where to refer these kids to…I think that it is really something that is missing and I think that if we have an overview of the referral possibilities, then we maybe ask more about it’. (FG1GP1)* In addition, parents noted that subsequent actions to help their child with its overweight should be non-committal. They prefer to let the subject sink in without being forced to take further steps. Though, parents would appreciate it if their GP remains involved by asking how things are going during a next visit. PNs and GPs expressed similar views that no role is seen for PNs in the treatment of overweight and obesity in children, because they are not trained nor have the right knowledge or skills to work with children. Moreover, it requires logistical adaptations to integrate PNs in this management.

## Discussion

### Main findings

This qualitative study explored the perspectives of GPs, PNs and parents about the role of general practice in the management of overweight and obesity in children. All three groups agreed that GPs should play a role in identifying and addressing overweight and obesity in children and should refer these children to suitable interventions. However, GPs experience several barriers when it comes to fulfilling this role. All parents, with and without a child with overweight, indicated that they are open to engage in a conversation about overweight when the GP is non-judgmental, interested and respectful. PNs saw no role for themselves in this management, a viewpoint that was supported by GPs.

### Strengths and limitations of the study

To the author’s knowledge, this is the first study that investigated perspectives of Dutch GPs, PNs and especially parents about the role of general practice in the management of overweight and obesity in children. Triangulation of these three perspectives provides unique insights in the needs and demands of GPs and parents which are important to take into account for the development of tools to support GPs in this management. Though some limitations must be addressed. Unfortunately, PNs specialised in youth were not willing to participate in our study. In order to get an impression about the possibilities to incorporate PNs in this management it was decided to include PNs with other specialisations. However, this may explain the finding that none of them saw a role since they do not have the experience nor skills to work with children. Next to that, the minority of the parents had a child with overweight. Only one of them had experience with a GP who noticed overweight in her child, in all other cases this was done by a youth doctor. In Dutch, a youth doctor is a specialist in the growth and development of children who often works at consultation clinics. Although we do not believe this cast doubt on the relevance of our findings since similar opinions and preferences were observed about the role of GPs between parents with and without a child with overweight in case excessive weight would be addressed in general practice. Another point of discussion is that no changes in the interview guides were made during the interviews, though no new topics arose during our interviews and therefore changing the guides was not needed.

### Comparison existing literature

In agreement with the Dutch GP guideline on Obesity, GPs recognised that they could play a role in identifying and addressing overweight and obesity in children. However, multiple GPs noted that they find this a task of youth doctors at consultation clinics where growth- and development measurements of children take place. However, children aged 4–12 years are limitedly monitored since they are only visiting these clinics at the age of 5 and 9 years [13]. Since most children aged 4–12 years visit their GP at least once during each life year, general practice is a unique setting to reach children and therefore offers a great opportunity for GPs to play a role in identifying and addressing overweight and obesity in children [5]. Moreover, all parents in this study preferred overweight in their child to be identified by their GP than the youth doctor because of the longitudinal doctor-patient relationship. Therefore, we recommend that GPs should play an active role in identifying and addressing overweight and obesity in children.

Fear for negative reactions of parents or children is a main barrier for GPs to address excessive weight in children, which aligns with literature [2,9,13,15]. Multiple GPs do not know how to initiate such conversations without being afraid to disturb the doctor-patient relationship or to negatively affect parents or children. However, this could be a misconception of GPs because this study and literature show that parents are open for weight-related conversations and prefer that overweight in their child would be addressed by their GP because of their doctor-patient relationship, GPs knowledge of the family history and the trusted setting [23]. But, all parents emphasised that the way how GPs address overweight is of great importance for the reaction of parents. Interestingly, the study of Strugiss et al. [24] showed that the confidence of GPs to initiate conversations and to discuss overweight with adult patients increased when GPs were provided with structured tools [24]. This might also apply in the case of initiating a conversation with parents and children. Furthermore, offering parents to discuss it in-depth during a follow-up consultation would be ideal since it provides both the opportunity to prepare for the conversation. We assume that specifically developed tools to initiate a conversation with parents and children and to propose a second appointment could improve GPs confidence and their ease to address and discuss overweight and obesity in children.

In this study, conflicting opinions were noted among GPs regarding bodyweight and height measurements in children who seem to be overweight. Reasons for not performing these measurements included GPs’ uncertainty about how to explain the need of it to parents and lack of clarity regarding BMI values of children. However, these measurements are of great importance because it provides insight in the seriousness of the child’s overweight to GPs and parents [20,25], since literature showed that many GPs and parents often under-recognise overweight and obesity in children [25,26]. In addition, the outcome of this measurement could support GPs to initiate a conversation with parents. For these reasons, in accordance with the Dutch GP guideline on Obesity we strongly recommend bodyweight and height measurement in children who appear to be overweight by GPs. In order to support GPs in conducting these measurements, we suggest to improve the accessibility of an easy to use BMI calculator specifically designed for children in general practice [27].

GPs noted that lack of clarity about referral options is another reason why they often do not address overweight in children during consultations. This may explain the low number of GPs referring children with overweight and obesity in the Netherlands [13]. Moreover, this observation strengthens the fact that GPs are in need of tools to assist them in referring children to appropriate interventions as already noted by multiple studies [13,17,28–30]. The development of an overview of appropriate interventions and referral options might be a possibility to increase GPs awareness about available interventions in order to support them in referring children with overweight and obesity.

### Implications for future research or clinical practice

In order to support GPs with the perceived barriers, future research should focus on evidence-based tools that can improve identification of overweight, help initiate conversations and provide referral materials. The required time for GPs to implement such tools during consultations should be evaluated as well. Additionally, education for GPs on cultural factors and related overweight is needed. It is important to investigate if a supportive tool is logistically feasible in clinical practice and whether the availability of the tool indeed improve the management of children with overweight and obesity in general practice.

## Conclusion

According to GPs, PNs and parental views, GPs are well placed to identify, address and refer children with overweight and obesity in general practice. Experienced barriers faced by GPs could be combated by offering tailored tools to support them performing these tasks.
